# Vascular and Osteological Morphology of Expanded Digit Tips Suggests Specialization in the Wandering Salamander (*Aneides vagrans*)

**DOI:** 10.1002/jmor.70026

**Published:** 2025-01-08

**Authors:** Christian E. Brown, William P. Goldenberg, Olivia M. Hinds, Mary Kate O'Donnell, Nancy L. Staub

**Affiliations:** ^1^ Department of Integrative Physiology and Neuroscience Washington State University Pullman Washington USA; ^2^ Goldenberg Film Arcata California USA; ^3^ Department of Biology Gonzaga University Spokane Washington USA; ^4^ Department of Biology Lycoming College Williamsport Pennsylvania USA

**Keywords:** arboreal, blood sinus, locomotion, mucous gland, toe

## Abstract

For over a century researchers have marveled at the square‐shaped toe tips of several species of climbing salamanders (genus *Aneides*), speculating about the function of large blood sinuses therein. Wandering salamanders (*Aneides vagrans*) have been reported to exhibit exquisite locomotor control while climbing, jumping, and gliding high (88 m) within the redwood canopy; however, a detailed investigation of their digital vascular system has yet to be conducted. Here, we describe the vascular and osteological structure of, and blood circulation through, the distal regions of the toes of *A. vagrans* using histology in tandem with live‐animal videos. Specifically, we sectioned a toe of *A. vagrans* at 0.90 μm, embedded it in Spurrs resin, and stained the tissue with toluidine blue. An additional three toes were sectioned at 10 μm, embedded in paraffin, and after sectioning and mounting, treated with Verhoeff and Quad stains. For living salamanders, we recorded real‐time videos of blood flowing within individual toes upon a translucent surface oriented both horizontally (0°) and vertically (90°) to simulate both prostrate and vertical clinging scenarios, then analyzed the image sequences using ImageJ. We found that the vascularized toe tips have one large sinus cavity that is divided more proximally into two chambers via a septum, and there are mucous and granular glands in the dorsal and dorsolateral integument of the digit tips. Live‐animal trials revealed variable sinus‐filling both within and between toes, seemingly associated with variable pressure applied to the substrate when standing, stepping, clinging, and climbing. We conclude that *A. vagrans*, and likely other climbing salamanders, can functionally fill, trap, and drain the blood in their vascularized toe tips to optimize attachment, detachment, and complex arboreal locomotion (e.g., landing after gliding flight). Such an adaptation could provide insights for bioinspired designs.

## Graphical abstract

Wandering salamanders (*Aneides vagrans*) exhibit exquisite locomotor control in the redwood canopy and have long digits with highly vascularized, bulbous, square‐shaped tips. The digital vascular network of *A. vagrans* constitutes a single connected sinus with two distinct chambers, and large mucous glands could work in tandem with the sinus chambers to optimize attachment and detachment. Blood fills, drains, and gets trapped within these sinus chambers as *A. vagrans* grips and detaches, with numerous functional implications.

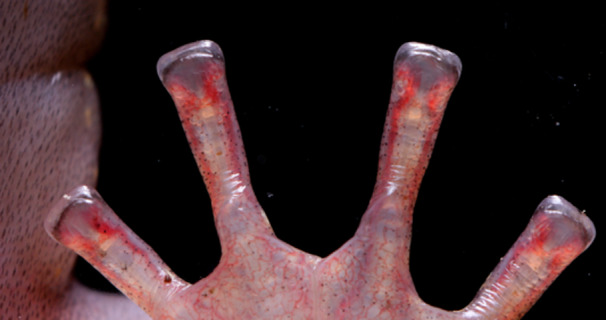

## 1 Introduction

Many taxa, including insects, arachnids, birds, mammals, reptiles, and amphibians, inhabit forest canopies around the world. Living in trees confers numerous advantages to arboreal animals, such as decreased competition for resources, increased foraging opportunities, and protection from ground‐dwelling predators (Kays and Allison 2001). However, arboreality poses locomotory challenges, such as climbing along variable substrates across a range of angles, gap‐crossing, and the avoidance of slipping or falling. Thus, arboreality is associated with a particular suite of fitness consequences ranging from displacement to injury and death (Cartmill 1985; Dudley et al. 2007; Jusufi et al. 2011; Humphreys and Ruxton 2019). As a result, many arboreal animals exhibit adaptive morphologies and behaviors that seemingly minimize these risks and help to keep them aloft (Dudley et al. 2007; Dudley and Yanoviak 2011).

The evolution of arboreality in salamanders and how it may relate to morphology is a subject of considerable interest, but making direct links between the evolution of salamander morphology and their occupation of specific microhabitat niches like rock faces, tree canopies, and cave walls has proved challenging. Evidence from arboreal and troglodytic salamanders suggests that foot shape has adaptive value, since species in these habitats show reduced rates of foot shape evolution compared to terrestrial species (Adams and Nistri 2010; Adams et al. 2017; Baken and Adams 2019; Salvidio, Crovetto, and Adams 2015) and there is evidence of allometric convergence in foot shape between these two climbing groups (Baken and Adams 2019). However, study of the evolution of foot shape has not supported the hypothesis of a distinct arboreal foot type or body shape (Baken and Adams 2019; Blankers, Adams, and Wiens 2012; Jaekel and Wake 2007). Clinging performance is positively correlated with foot centroid size and contact area when clinging to smooth surfaces, and negatively correlated on rough surfaces (Baken and O'Donnell 2021). Some reasons for this ambiguity in the relationship between form and function may be due to the high overall prevalence of climbing behavior in salamanders in general (McEntire 2016), the ease with which many salamander species can achieve attachment across substrates with variable roughness, wetness, and angle (O'Donnell and Deban 2020a, 2020b; Hanna et al. 2022; Wang et al. 2016), and the evident overlap in morphology and behavior between species which are obligately arboreal and those that are facultative climbers (Baken and Adams 2019; Blankers, Adams, and Wiens 2012; McEntire 2016; O'Donnell and Deban 2020a, 2020b).

One group of arboreal amphibians that has received considerable attention in this regard is the climbing salamanders of North America (genus *Aneides*). Despite not all the species being arboreal, the clinging and climbing ability of several species of *Aneides* has fascinated biologists for no less than 125 years (Ritter and Miller 1899; Diefenbacher 2008a; O'Donnell and Deban 2020a). The climbing species of *Aneides* share a number of traits that strongly suggest specialization for climbing: prehensile tails, relatively long limbs and digits, and large, square‐shaped toe tips, with some species also being dorsoventrally flattened (Petranka 1998; Stebbins 2003). The square‐shaped toe tips are especially intriguing, as they are highly vascularized, housing large sinuses that are easily seen as bright red “lakes of blood” in living specimens (Ritter and Miller 1899). Early accounts describe a large blood sinus present on each side and near the distal end of each toe in *Aneides lugubris* (see Figure 3 in Ritter and Miller 1899), hypothesized to be important for gas exchange and the oxygenation of the distal digits, and going so far as to say that the toes assume the function of external gills (Ritter and Miller 1899). However, similar sinuses are present in most, if not all, salamanders (Noble 1925), and these sinuses do not correlate with other morphological features which could facilitate cutaneous oxygenation, such as thin epidermal layers over the blood vessels (Noble 1925). Furthermore, while it is possible the sinuses contribute to gas exchange in some capacity, empirical evidence for this hypothesis is lacking. Other hypotheses regarding the digital vascular system of *Aneides* suggest that it prevents desiccation or electrolyte imbalance (Brown 1972; Burggren and Moallf 1984), but these too lack empirical evidence.

Perhaps the most detailed anatomical description of the blood sinuses examined stained histological preparations of the vascularized toe tips of *Aneides aeneus* (Diefenbacher 2008b), a salamander primarily found in rock crevices but known to climb trees (Petranka 1998; Stebbins 2003). Based on comparisons of *A. aeneus* to the sympatric, non‐climbing species, *Plethodon kentucki* and *Plethodon glutinosus*, Diefenbacher (2008b) suggested that features of the toe morphology of *A. aeneus*, such as the thickened integument and secretions from its glandular derivatives, serve as a friction‐based attachment mechanism, allowing the distal portion of the digit to deform and flexibly grip a rough substrate. Interestingly, *Aneides vagrans* excels in clinging to rough vertical and overhanging surfaces, even being able to support its body weight on a single digit (O'Donnell and Deban 2020a, 2020b), which supports Diefenbacher's attachment hypothesis. Using live specimens, we test Diefenbacher's (2008b) attachment specialization hypothesis regarding form and function of the vascularized toe tips of *Aneides*, focusing on *A. vagrans*.

Wandering salamanders, *A. vagrans* (Jackman 1998), live in the crowns of old‐growth coast redwood trees (*Sequoia sempervirens*) as well as terrestrially, under the bark of tree stumps and downed logs and under rocks (Petranka 1998; Stebbins 2003). In the canopy, they take refuge in complex arrays of epiphytic fern and humus mats, but have also been observed moving vertically along the trunk and branches as high as 88 m off the forest floor (Sillett 1999). At such elevations within the coastal fog, these salamanders can conduct their entire life cycle without leaving the canopy (Spickler et al. 2006). To locate prey and mates, avoid desiccation, or just wander about the canopy, *A. vagrans* has a number of adaptations for arboreal locomotion, including an altered climbing gait (Aretz, Brown, and Deban 2021), high clinging performance (O'Donnell and Deban 2020, 2020b), a unique jumping style (Brown and Deban 2020), and appropriately oriented body surfaces (i.e., tail, trunk, and feet) that facilitate air‐righting, parachuting, and gliding while airborne (Brown et al. 2022). Thus, *A. vagrans* is the most arboreal species within *Aneides*, and arguably the most arboreal species of all salamanders. However, the digital vascular system of this highly arboreal amphibian remains undescribed. Here, we describe the histology of, as well as the dynamics of blood circulation through, the toes of *A. vagrans* using histological approaches in tandem with live‐animal videos, our goal being to elucidate the functional anatomy of the digital vascular system. Given that closely related and morphologically similar species of *Aneides* have large blood sinuses at the digit tips, we hypothesized that *A. vagrans* would possess them as well; furthermore, we hypothesized that *A. vagrans* regulates blood circulation through the digital vascular system and its associated large sinuses while locomoting, perhaps rapidly expanding and contracting their toe tips to optimize locomotion or attachment. Here, we describe the digital vascular system of both preserved and living specimens of *A. vagrans*.

## 2 Materials and Methods

In January 2022, we collected three *A. vagrans* specimens from Humboldt and Del Norte counties, California under CADFW Scientific Collection Permit #13153 (to CEB). We limited our sample size to minimize impact on wild populations, taking only the minimum number to reasonably test our hypothesis. We video‐recorded the movement of toe tips of all three live salamanders as described below and used fluid‐preserved specimens of *A. vagrans* from our private collections (CADFW Scientific Collection Permit #13153 to CEB) (*N* = 3; two males, one female) for histological analyses.

### 2.1 Histological Preparations

We prepared slides using digits from three *A. vagrans* specimens (*N* = 3; two males, one female) that were originally fixed in 10% neutral‐buffered formalin and preserved in 70% ethanol. For each specimen, we severed the third toe from the hind foot (Digit III) with a triangular cut at the base to indicate the proximal end. Tissues were decalcified in Cal‐Ex (Fisher Scientific, Waltham, MA, USA), and embedded in paraffin wax. We sectioned the digits transversely at 10 μm on a Leica Jung 2035 Biocut rotary microtome and mounted samples onto glass slides. We used both the Verhoeff (most slides) and Quad stain (approximately every 10th slide) (Floyd 1990; Staub and Paladin 1997) to visualize toe morphology. To identify mucous (vs. granular) glands, we used the Quad stain, which uses the periodic‐acid Schiff (PAS) reaction to identify neutral carbohydrates, alcian blue (pH = 2.0) to identify mucopolysaccharides, naphthol yellow to identify proteins, and Gill's hematoxylin to identify nuclear DNA. The Verhoeff stain was used to visualize blood cells and the extent of the blood sinuses, by staining elastin (walls of arteries, sinuses) and nuclei black, collagen red, and cytoplasm yellow. The length of the blood sinus was approximated by counting all sections that included components of this structure (any tissue sections lost while sectioning were included in the estimate). We examined all slides using a light microscope (Leica DMC) and took all photomicrographs using a microscope‐mounted EOS Rebel 5 camera (Canon, Melville, NY, USA).

One additional digit (Digit III) from the alternate hind foot of one of the three specimens was cross‐sectioned at 0.90 μm after being embedded in Spurrs resin at the Franceschi Microscopy and Imaging Center (FMIC) at Washington State University (Pullman, WA, USA) and stained with 1% aqueous toluidine blue (stains nuclei blue) for 30–60 s on a hot plate.

These histological approaches allowed us to describe the anatomy of the digital vascular system and this description was used to inform our interpretations of the live‐animal trials. Likewise, the live‐animal trials were used to inform and confirm any observations made during the interpretation of the histological sections.

### 2.2 Live‐Animal Trials

**Figure 1:**
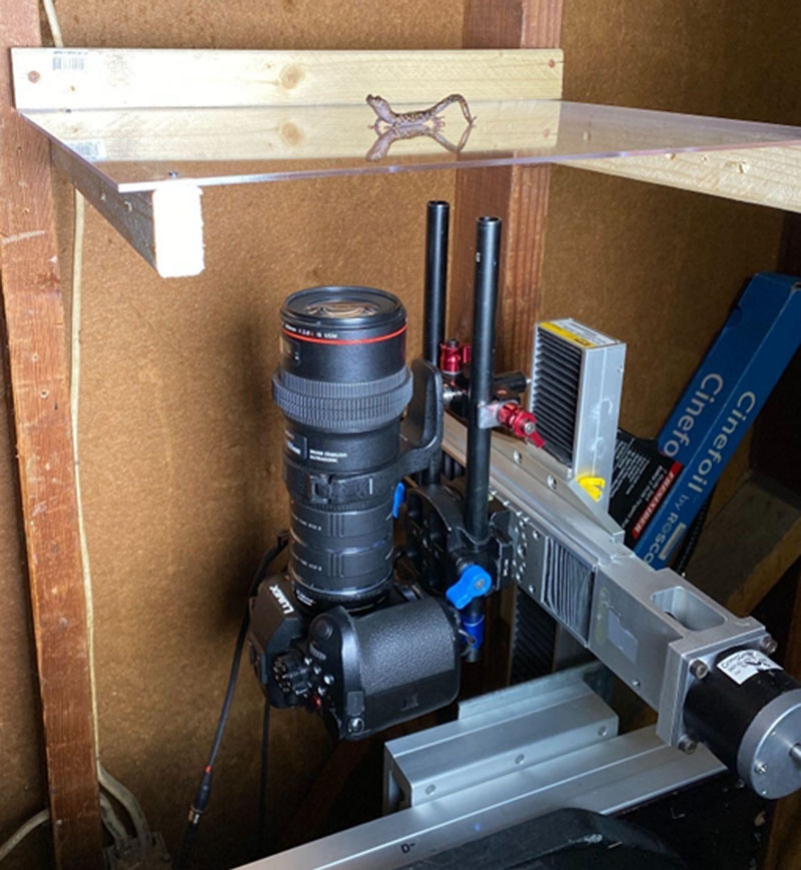
Custom built salamander‐viewing platform and camera slide used to image live *Aneides vagrans*. The viewing platform can be positioned vertically or horizontally (as shown here) and secured during trials. The viewing platform was made of clear acrylic such that images from the ventral view can be easily captured, the latter being the most expansive single view for visualizing the entire digital vascular system.

We recorded real‐time videos of blood circulating through the digital vascular system of the toes of living salamanders using a Panasonic GH6 camera (Panasonic, Newark, NJ, USA) equipped with a 100 mm Macro lens (Canon, Melville, NY, USA) attached via a full‐frame to micro four‐thirds lens adapter (Metabones, Vancouver, Canada). The resulting magnification was 1.4× (i.e., 1× lens magnification × 2× camera sensor crop factor × 0.7× lens adapter magnification). Focus was trained on the toe tips of the hind limbs, assisted by a custom‐built platform and camera slider (Figure 1), wherein the camera was attached to three integrated motorized sliders that allowed for precise control over the lateral (*x*), vertical (*y*) and focal distance (*z*) positioning of the camera via a wired joystick control.

**Figure 2:**
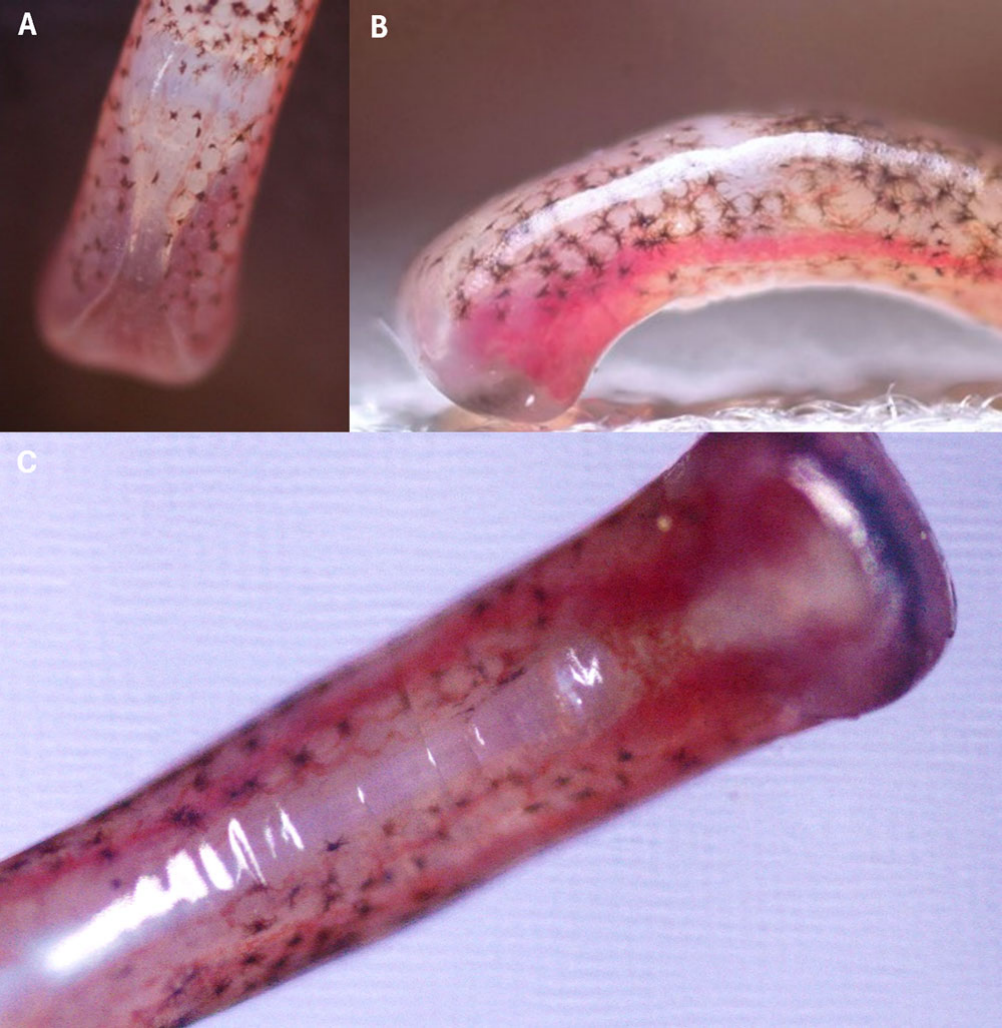
Still frame images of Digit III, the middle toe, of a live *Aneides vagrans*. Images were taken with the salamander in a neutral posture on a flat surface from (A) dorsal, (B) lateral, and (C) ventral views. These images show the digital sinus and the bifurcated terminal phalanx of a living salamander; the structure of these features was corroborated by our histological preparations, shown in Figures 3 and 4.

We placed individual salamanders on a viewing platform that consisted of a sheet of clear acrylic (25 cm × 40 cm, 3.175 mm thick) and positioned the platform such that the ventral surface of the toes was visible to the camera (Figure 1). We conducted trials with the viewing platform oriented horizontally (tilted 0°) as well as vertically (tilted 90°) to simulate both prostrate and vertical clinging scenarios. Once optimal focus was achieved on the maximum number of digits, we started recording and continued until the salamander took a step forward causing the toe to leave the frame of view, henceforth referred to as *toe off*. Digits are referred to as I–V, with I being the most medial and V the most lateral. We determined it inappropriate to analyze the tips of Digit I, the highly reduced, medial‐most digit, because it often required a different field of focus compared to Digits II–V, thus, it was more difficult for us to visualize blood flow (Figure 2). Furthermore, we attempted to record blood flow of entire hind limbs and their associated digits but were limited by the range of focus possible at such a small scale.

We recorded and analyzed six trials at 90° and six trials at 0° for all three salamanders, including only trials wherein the salamander stayed motionless for at least 10 s before toe off to provide a sufficient window of time for analysis. Across the three individual salamanders, we successfully recorded two to four toe tips per trial (*n* = 12 trials per individual, six vertical and six horizontal), including Digits II–V of both hind feet for all three salamanders (i.e., 24 toe tips), for a total of 98 toe tip video sequences, 52 vertically oriented and 46 horizontally oriented, for analysis.

We first assessed the image sequences qualitatively and recorded instances of blood filling or draining as well as instances of heterogeneous blood distribution within and between toes (e.g., more blood in the right chamber of the sinus compared to the left within a single toe, or more blood in a given toe tip compared to the others on that foot). Next, we converted the .mov files to image sequences and isolated the images at 300 frames (10 s), 30 frames (1 s), and 1 frame (immediately) before the digit's disengagement from the substratum; these categories were meant to represent blood sinus status at latent, intermediate, and engaged toe tip states, respectively. We used the Oval tool in ImageJ (National Institutes of Health, Bethesda, MD, USA; Rasband 1997–2023) to create and shape custom polygons that encompassed the entire square‐shaped toe tip (one polygon per toe), including both sinus chambers and the connecting vessels that occur at the pinch‐point where the sinus chambers are closest together (Figure 2C). We then used the RGB color channel in ImageJ to quantify the minimum, mean, and standard deviation of red pixels present within, henceforth referred to as *digital redness*, for all trials and treatment groups. To control image white balance and preclude any color cast that might bias the digital redness measurements, we only analyzed the change in digital redness within trials; moreover, we never compared the raw values for digital redness between trials nor used those values in our statistical analyses.

**Figure 3:**
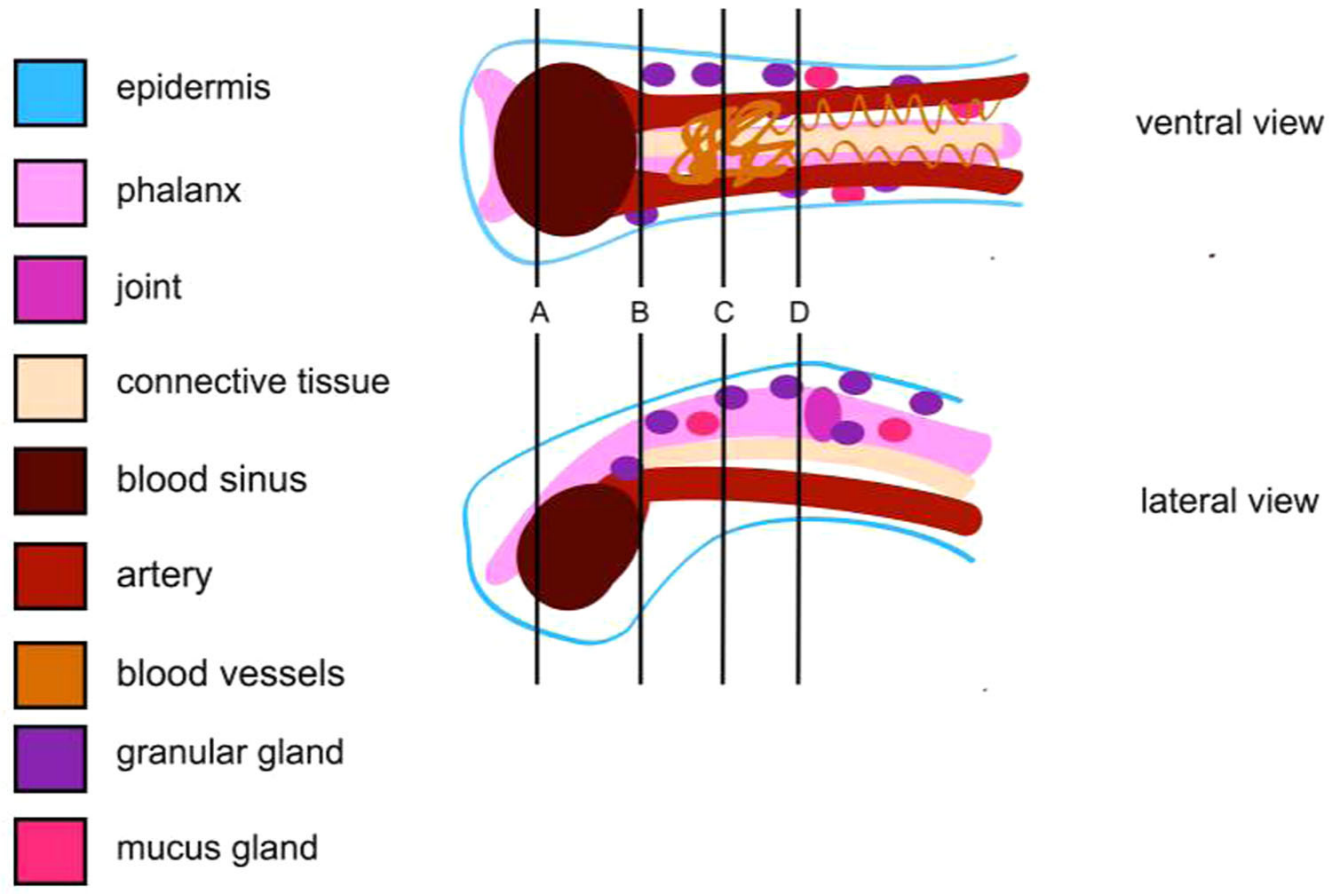
Detailed schematic representation of the toe tip of *Aneides vagrans.* Schematic representations are shown in ventral and lateral views. Schematics show the epidermis (blue line), terminal phalanx (light pink), joint (magenta), connective tissue (beige), blood sinus (brown), artery (red), blood vessels (orange‐brown), granular glands (purple ovals), and mucus glands (pink ovals). Lines drawn through the toe at four distinct points along the toe tip (A–D) indicate the points at which the cross‐sections featured in Figure 4 were taken.

We analyzed change in digital redness in RStudio Team (2020) using a mixed effects model with toe, orientation, and seconds before toe off as categorical fixed variables, individual salamander as a random variable, and change in digital redness as the dependent variable. We selected the simplest model that best fit the data based on AIC. Notably, the model did not test for significant differences in redness but rather for significant predictors of change in redness and measured what degree of change in redness is predicted by each variable. For one exemplary image sequence taken from the lateral perspective, we also used the distance tool in ImageJ to measure the diameter of the main lateral artery of the digital vascular system as well as the breadth of the entire toe tip at its widest point. The effect of blood perfusion on digital morphology could only be observed from this lateral perspective, which proved difficult to replicate; therefore, we only used the highly replicable ventral perspective when imaging the toes for statistical analyses. We provide a schematic representation of the toe tip that is informed by both the histological slides and the live‐animal trials (Figure 3) to better aid the visualization of the digital vascular system of *A. vagrans* from both this lateral and ventral views.

## 3 Results

### 3.1 Histological Preparations

#### 3.1.1 Distal‐Most Region

**Figure 4:**
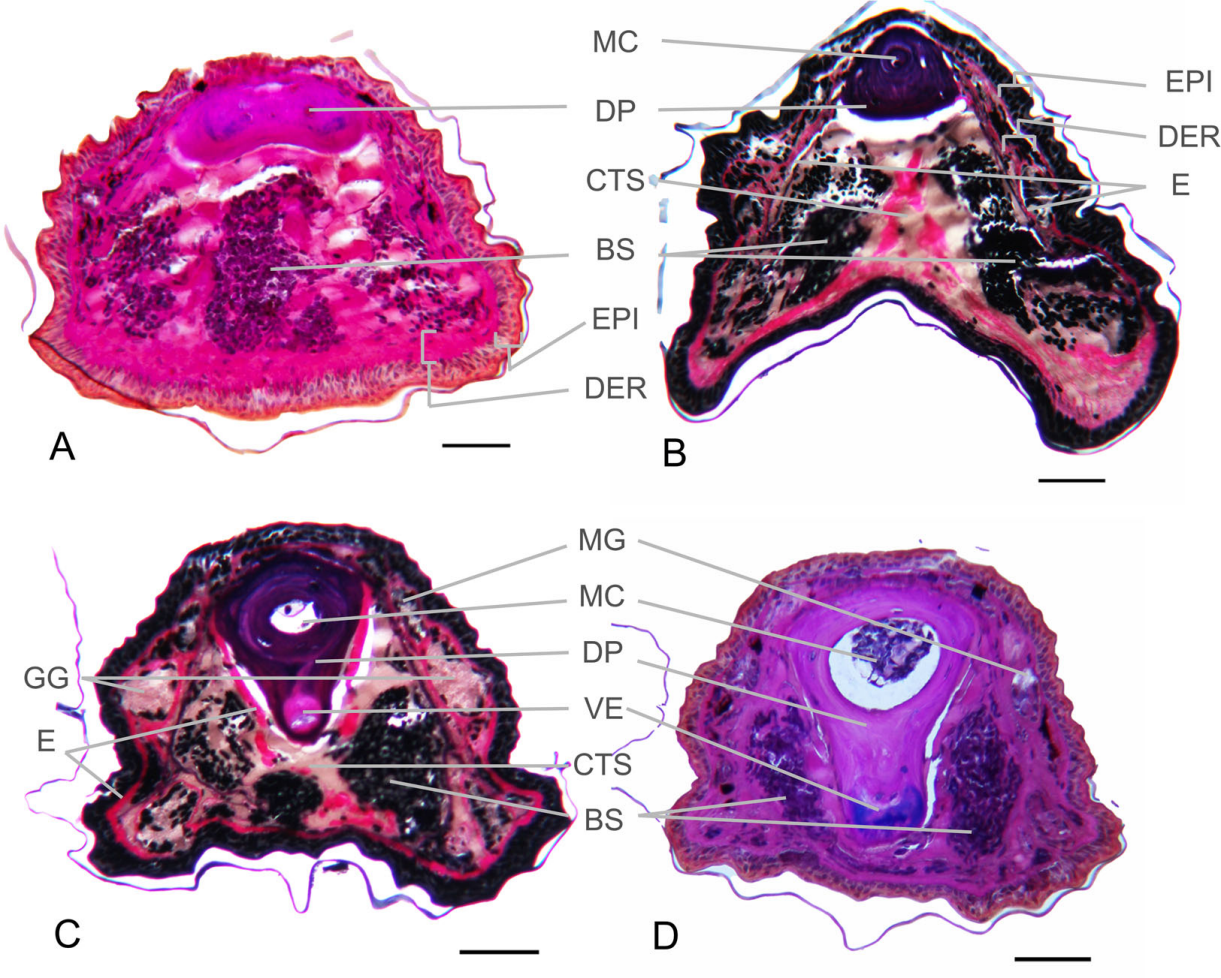
Histology of *Aneides vagrans* toe. Photomicrographs of 10 μm (A, B, C, and D) cross sections of Digit III of the hind foot. Photomicrograph panels A and D represent slides stained with Quad stain, while photomicrographs C and D are stained with Verhoeff stain. Series A through D represent slides moving from most distal (A) to most proximal (D) along the distal phalanx before the most distal interphalangeal joint. Anatomical components visible: BS, blood sinus; CTS, connective tissue septum; DER, dermis; DP, distal phalanx; E, tissue staining positive for elastin; EPI, epidermis; GG, granular glands; MC, medullary cavity; MG, mucous glands; VE, ventral extension of the distal phalanx. Scale bars are 100 μm.

At its most distal region (Figure 4A), the general shape of the toe in cross section is round and bifurcation of the distalmost phalange is evident, although it is unclear from our sections whether the distal phalange actually splits into a Y‐shaped process or stays more spatulate in shape. The curved and bifurcated or spatulate distal phalanx is consistent with that described for other species of *Aneides* (Diefenbacher 2008a, 2008b; Wake 1963; Wake, Wake, and Wake 1983). We observed dark nuclei from the Verhoeff stain indicating blood cells in the sinus, shown here as a large sinus at the tip of the toe (Figure 4A). All four toes examined showed a large sinus in the distal region of the toe, with varying amounts of connective tissue located within (Figures 4A and S1). The sinus is largest at the tip of the toe, where the sinus fills the space ventral to the distalmost phalanx (Figure 4A). The sinus terminated an average of 73 ± 15 μm from the distalmost point of the toe. The average total sinus length is 673 ± 84 μm.

The toe in cross‐section becomes more involuted (horseshoe‐shaped) more proximally, with ventral projections lateral to the involution (Figure 4B). Observations on preserved specimens indicate this horseshoe‐shape is an artifact of fixation. A medullary cavity within the phalange is evident (Figure 4C,D). More proximally, the digital sinus is subdivided into two compartments (Figure 4B–D).

#### 3.1.2 Proximal Sinus Region

Both the M. extensor brevis distalis and tendon flexor digitorum longus attach to the distal phalanx. The ventral side of the distal phalanx in more proximal sections of the distal phalanx extends downward into a ventral projection (Figure 4C,D). Proximal to the terminal region of the sinus, a connective tissue septum separates the sinus into two compartments (Figure 4B) that flank the ventrolateral aspects of the phalanx (Figure 4B–D). It appears that although the two compartments of the sinus are separated, there is still a connecting channel between the two that permits blood flow between them (Figure 2C; Movie S2). The partially‐segregated sinus chambers further separate from each other more proximally (Figure 4C,D).

Proximal to their greatest width, the sinus chambers diminish in diameter to that of blood vessels by the point at which the distalmost and penultimate phalanges articulate. An exception was observed in one specimen wherein the sinus extended slightly proximal to the distal‐most joint, showing there is some variation in its extent.

Mucous and granular glands are present along the dorsal‐dorsolateral surface primarily proximal to the distal tip of the toe (Figure 4B–D). Fuchsin Schiff positive granular glands, indicating the presence of neutral carbohydrates, are also present on the lateral surface (Figure 4D).

### 3.2 Spurrs Resin Embedded Specimen

The single specimen embedded in Spurrs resin at FMIC reveals a single, undivided sinus cavity in the distalmost region of the toe (Figure S1), slightly distal to Figure 4A in the region where the phalanx is wider and more spatulate. This specimen also shows a thicker epidermis on the ventral side compared to the dorsal side of the toe tip, and several glands in the dorsal and dorsolateral integument (Figure S1).

### 3.3 Live‐Animal Trials

**Figure 5:**
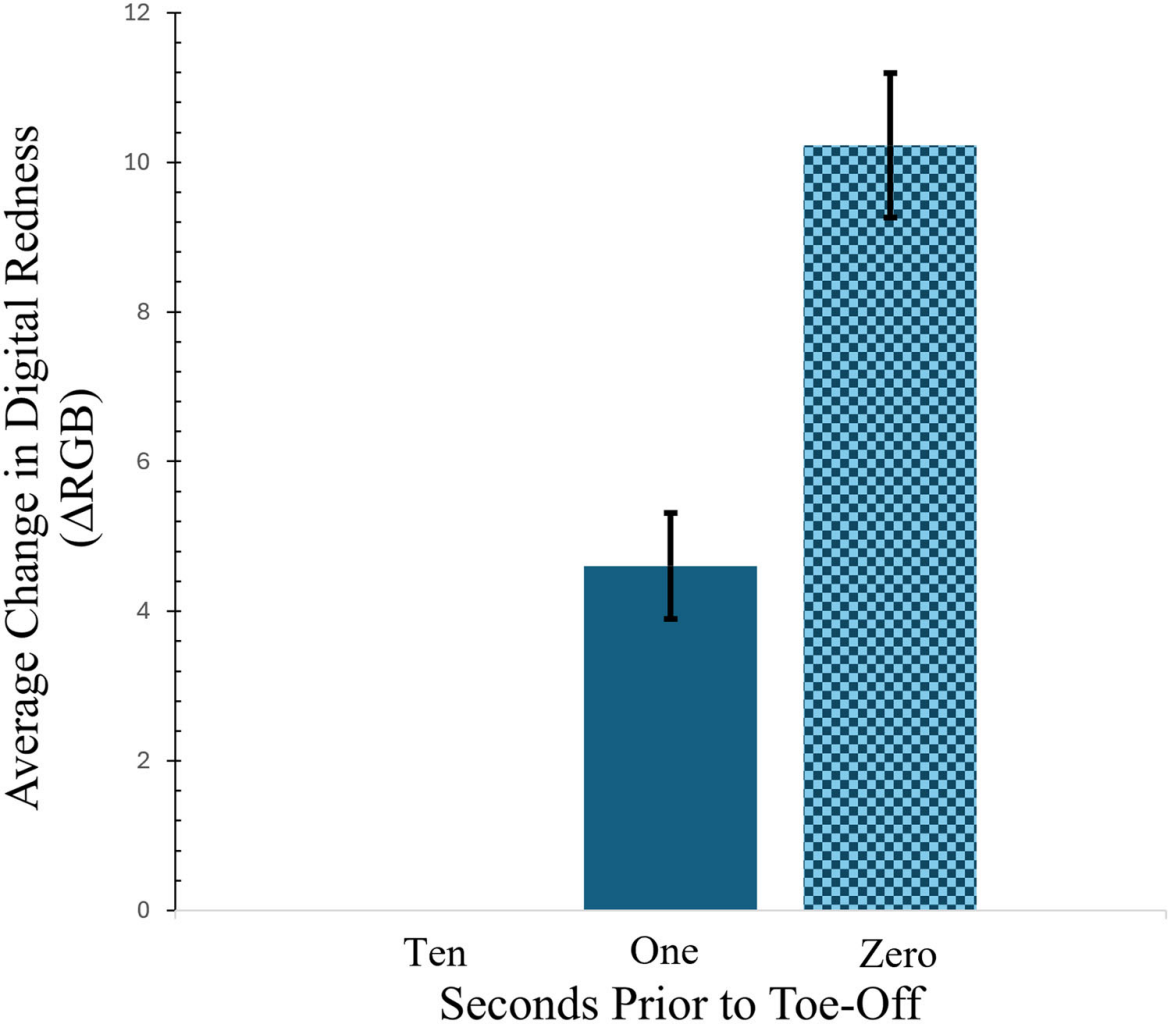
Bar graph comparing the average change in redness (blue bars) from images of the digital sinuses of *A*. *vagrans* under various locomotory conditions. On average, the digital redness is highest in the last frame before toe off and lowest at 300 frames, or 10 s, before toe off. Standard deviations are shown as error bars (black).

The digital vascular system of *A. vagrans* was clearly visible during standing and walking on our imaging platform in both ventral and lateral views (Figure 2). We found that the digital vascular system of *A. vagrans* showed rapid filling of the large sinuses at the tips of the toes and movement of blood within them. Blood appeared to form bifurcated pools more distally, with a strong central connection more proximally, proximal to the distalmost interphalangeal joint (Figure 2C). This contrasts with the histological evidence, which shows a centrally connected sinus distally (Figure 4A), suggesting perfusion may change the shape and appearance of the sinus. Another plausible explanation for this discrepancy could be that the more distal connection between sinus chambers seen in histology exists deeper than can be seen in living salamanders with a camera; although the skin of the toes is translucent, other soft tissues could obstruct the view. Quantity of blood, as measured by increased digital redness (Movie S1), changed, particularly at the time of disengagement from the substratum, revealing rapid increases in redness leading up to and at the time of toe off (Figure 5).

This observation was supported quantitatively by our mixed effects model. The best fitting model (Formula: MeanRedness ~ (1| Individual) + Orientation + SecondsPrior) included individual as a random effect, and the platform orientation (0° or 90°) and seconds before toe off (10 s, 1 s, or last frame before toe off) as fixed effects. The model's overall ability to explain variation in the change in digital redness was moderate (conditional *R2* = 0.14) but it suggests that orientation (*Effect Estimate* = −16.351; *t* = −5.743; SD = 2.847) and seconds before toe off (*Effect Estimate* = 8.812; *t* = 2.546; SD = 3.462) have the strongest and the second‐strongest effects on change in digital redness, respectively, although there was considerable variation in change in digital redness between individuals (*Variance* = 26.416; SD = 5.140). The negative effect of orientation on redness suggests less blood is present in the toe tips when clinging vertically, while the positive effect of time before toe off shows that blood is moving into the toe tip as the foot begins to disengage from the substratum. Overall, across both orientations and all three time points, extensive variation in change in digital redness was observed among individuals and between trials (Figure 5).

**Figure 6:**
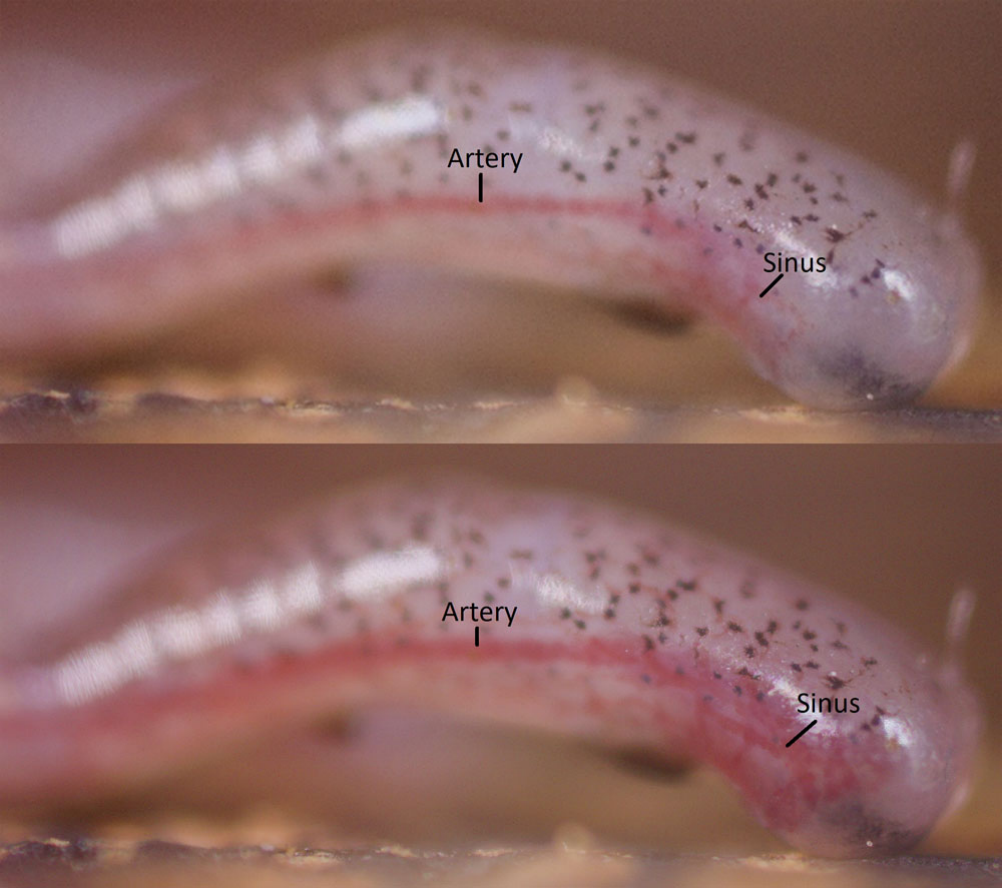
Still frame images of Digit III, the middle‐most toe, of a live *Aneides vagrans* when (A) drained of (top) vs. (B) engorged (bottom) with blood. Both (A and B) show the same toe of the same salamander during the same experimental trial, the images being separated by ~25 s. The artery supplying the digital sinus increased in visible width by ~23 μm, or ~25%, and the toe tip extended proximally on the ventral side, with distance measurements at the longest section of the toe tip increasing proximally by ~45 μm, or ~5%. See Movie S2.

For one especially clear and representative trial (Figure 6, Movie S1) that captured the entire sinus and the artery supplying it (Figure 5) in a lateral perspective (but could not be reliably replicated), we were able to calculate the total time required for complete filling of the sinus to be ~25 s, during which the lateral digital artery supplying the sinus increased in visible diameter (measured dorsal to ventral) by ~23 μm, or ~25%, and the ventral surface of the toe tip expanded by ~45 μm, or ~5% at its widest point (Figure 6). The toe tip appeared to engorge proximally on the ventral surface, although any expansion of the toe tip laterally towards the camera or hidden on the far side of the toe could not be observed when imaged from this lateral perspective.

The two large blood sinus compartments of each toe tip are connected, and blood can flow in either direction between them in their proximal regions adjacent to the ventral integument (Movie S2). This connection point seems to correlate with the distalmost interphalangeal articulation close to the base of the square‐shaped digit tip itself. In addition to observations of the sinuses and the overall digital vascular system, we observed the terminal phalanx (Figure 2A) and numerous pleats on the ventral epidermis of the toes and toe tips of *A. vagrans* just before toe off (Movie 3).

## 4 Discussion

In this investigation, we describe the morphology of the square‐shaped toe tips of the wandering salamander (*A. vagrans*), using both live animal imaging and histological preparations. Here, we discuss intriguing features of the toe tip morphology, compare our findings with the morphology and function of relevant species, and propose some hypotheses and future experiments to investigate their possible biomechanical functional implications. The limitations in imaging blood flow in a freely moving animal require further experiments and performance analyses to directly assess the relationship between digital vascular activity and surface attachment or locomotion.

Evidence suggests that salamanders can implement a variety of attachment strategies to successfully locomote in arboreal or scansorial habitats, which vary depending on the substrate angle, surface roughness, surface wetness, perch diameter, and other features (Hanna et al. 2022; O'Donnell and Deban 2020a, 2020b; Wang et al. 2016). Animals locomoting on challenging surfaces are partly dependent on their ability to generate reversible attachment to the surface. While many attachment strategies exist, those most relevant in the context of salamander locomotion are friction, adhesion, and gripping; aspects of digit histology and live animal performance suggest ways in which *A. vagrans* digital features may act to promote all three, as needed for the specific demands of the substrate. Our findings suggest the digital vascular system could be used to increase toe surface area, increase toe pad flexibility, or decrease the force needed to detach from the substrate, possibilities that are not mutually exclusive. Positioning of mucous glands on the dorsolateral surface of the distal region of the toe, close to the distalmost interphalangeal joint but proximal to the expanded toe tip, could ensure optimized mucus distribution to prevent drying or flooding of the toe pad. Curved distal phalanges provide favorable articulation mechanics and likely improve interlocking and gripping capability on rough surfaces. Each of these morphological features is discussed here, and will be used to inspire specific functional testing in the future.

### 4.1 Curved Distal Phalanx

Distal phalanges are recurved, with a bifurcation at the toe tip, although it is not clear whether the distal phalange actually splits into a Y‐shaped process or stays more spatulate in shape. The curved and bifurcated or spatulate distal phalanx has been described in other species of *Aneides* (Diefenbacher 2008a, 2008b; Wake 1963; Wake, Wake, and Wake 1983), and forked phalanges occur in a genus of geckos, which also lacked claws (Dixon and Kluge 1964).

Closer to the joint, the distal phalanx has a ventral process (Figure 4C,D) which is the eventual site of attachment for the digital musculature, and the attaching tendon appears to be visible through the skin in some ventral images of the foot (Figure 2C). Differences in the size of this ventral process have been documented among several plethodontid species encompassing a range of microhabitat preferences (Wake 1963), but whether these differences correspond to any functional difference in grip strength or digital control is not known.

### 4.2 Blood Sinus

The digital vascular system of *A. vagrans* does indeed include a large blood sinus spanning from the distalmost interphalangeal joint to the tip of each digit, similar to other species in its genus (Ritter and Miller 1899; Diefenbacher 2008a; Raygoza 2018). Blood can efficiently fill and drain the sinus. We show here that the digital vascular system of *A. vagrans* includes connections between the ventrolateral sinus compartments in the more proximal regions of the toe tip allowing for the observed rapid changes in the amount and location of blood as it moves from one sinus compartment of the square‐shaped toe tip to the other (Figures 2, 4, and 6; Movie S1). Considering our live‐animal and histological observations, we posit that the digital vascular system of *A. vagrans*, and likely other congeneric climbing salamanders, constitutes a single sinus with two distinct, though connected, chambers more proximally.

Animals in the live trials were recorded with blood being distributed unevenly between toes, between chambers of the blood sinus, and with regard to the amount of blood within the sinus between trials, substrate angles, and over the course of a single trial leading up to toe off.

Increase in redness indicates increased perfusion before toe off, and inspires questions about the effects of blood movement in these structures. For some species, swelling of the toe pad structure increases the curvature of the toe pad, decreasing the contact area as the stiffer arc pushes away from the substrate at the edges (Dening et al. 2014; Dai and Gorb 2009). In *A. vagrans*, the timing of the movement of blood into the toe directly before toe off suggests a role in detachment; detaching from the substratum with minimal energy expenditure while keeping the toe tip in pristine configuration for its next use is critical.

### 4.3 Other Sources of Variation in Redness

While proximity in time to toe off and orientation were predictors of change in redness (perfusion) of the toe within trials, the experimental methods precluded quantifying absolute perfusion across trials with consistency. Some of the variation in blood distribution may be due to behavioral or environmental factors. In fact, we anecdotally noticed that the salamanders needed a warm‐up period after being taken out of their chilled housing containers (~10°C) and placed onto the room‐temperature (~20°C) viewing platform, suggesting that temperature could be an important factor in future investigations. Some of this variability in change in digital redness could be a product of different body postures between trials lending to variable shadows cast upon the toes. For this reason, the analysis focused on the overall positive or negative effect of orientation and time before toe off rather than the estimated effect size. Change in digital redness in each toe was not highly variable within trials and followed a consistent and significant trend of increasing blood presence in the sinus closer and closer to toe off.

### 4.4 Mucous Glands

Mucous glands were identified on the dorsal‐dorsolateral surface of the toe. In contrast to these findings in *A. vagrans*, for *Bolitoglossa*, the foot has a smooth ventral surface prolifically endowed with mucous glands (Green and Alberch 1981). Wet adhesion is the most likely method of attachment on smooth substrates for salamanders because of their high cling performance on vertical and overhanging surfaces (O'Donnell and Deban 2020, 2020b). Their ventral surface (trunk, limbs, feet, and tail) is coated with a thin layer of mucus, and their toe tips have been shown to be making intimate contact with smooth substrates (O'Donnell and Deban 2020, 2020b). Small amounts of fluid are shown to improve attachment on moderately rough surfaces in other plethodontid salamanders (O'Donnell and Deban 2020b). However, the position of these glands seems to indicate that direct secretion of mucous to the ventral surface of the toe tip is not required for effective clinging or locomotion.

For organisms that use mucus‐based wet adhesion, accruing a thin layer of mucus on the ventral surface of the toe pad can ensure a large area of contact between the organism and the substrate, even on rough surfaces, because mucus fills in small gaps between the organism and the substrate (Federle et al. 2006). When the mucous layer is too thick, however, it can instead take on a lubricating effect which prevents the formation of any direct contact, reduces friction between the organism and the substrate, and increases the animal's vulnerability to shearing forces. Evidence suggests that tree frog toe pad structures optimize the distribution of mucus into thin layers, or even allow direct contact between the toe pad and surface at some locations, to maximize frictional and adhesive forces (Barnes, Oines, and Smith 2006; Smith et al. 2006).

The few available investigations of salamander toe surface morphology indicate that the ultrastructure of the integument of plethodontid toes is generally smooth (Green and Alberch 1981) lacking the abrasive‐resistant cell structures seen in tree frogs (Green 1979). Some newts, however, have toes with an integumentary ultrastructure consisting of a dense array of nanopillars surrounded by a network of small channels (Wang et al. 2016). We observed prominent pleats forming on the toe tips of living *A. vagrans* in some trials, particularly in instances close to the initiation of toe off (Movie S3). Their function in relation to surface attachment, if any, is likely dissimilar to that of the large‐scale structures seen on gecko and tree frog toe pads. More evidence is needed to investigate any functional attachment role of mucus or the skin‐substrate interaction in relationship to the observed variation in blood sinus perfusion.

### 4.5 Blood Flow Control Hypotheses

Taken together, our observations and analyses suggest that the digital vascular system of *A. vagrans* functions mechanically to confer control over the amount of blood directed to and held within the central or lateral regions of the blood sinus, and may aid arboreal attachment and locomotion more generally. The anatomy and relationship between the components of the digital vascular system in *A. vagrans*, discussed above, seem to align with, rather than refute, the hypothesis originally proposed by Diefenbacher (2008b) that the sinuses of *Aneides*, *A. aeneus* in this case, could function as a surface attachment mechanism. Varying the contact area, flexibility, and stiffness of the toe tip in contact with the surface could be used to enhance either adhesive or frictional forces.

In support of the mechanical control hypothesis is the rapidity with which the sinuses fill with blood, with changes in digital redness being observed in less than 1 s, and the corresponding increase in vascular dilation. Similar vascular features exist in other clinging and climbing amphibian species, for example, *Litoria* tree frogs. These arboreal frogs also have a blood sinus ventral to the terminal phalanx of the toes, although it is situated more centrally within the toe compared to the digital sinus of *A. vagrans* (Barnes et al. 2011; Scholz et al. 2009), perhaps providing a toe surface with enough flexibility to excel at clinging to roughened surfaces, as reported for *Aneides* (O'Donnell and Deban 2020b). Tokay geckos (*Gecko gecko*), world‐renowned masters of arboreal locomotion have a digital vascular system similar to that of *Aneides*. The interactions between the wide lateral arteries, the reticular networks, and the sinuses, as well as smooth muscle bands in both the sinus and the distal drainage vessel walls, control the pressure within the system to alter the deformation and compliance of the toe pads (Russell 1981). Sequential filling and draining of the large sinuses in the toes of some frog species (Krogh, Harrop, and Rehberg 1922), and active modification of pad shape and stiffness by varying blood pressure, has been proposed for tree frogs (Langowski et al. 2018).

Beyond compliance for attachment, the blood sinus could fulfill other roles. The integument of tree frog toe pads has been shown to be one of the softest biological structures in existence (effective elastic modulus of 4–25 kPa) and may possibly serve as a shock‐absorbing material during arboreal locomotion (Barnes et al. 2011). *A. vagrans* leaps and glides from the world's tallest trees and would likely benefit from similar shock‐absorbing properties endowed by a gradient of stiffness at the toe tip.

If blood flow control mechanisms are at work in *A. vagrans*, blood moving into the digital sinus could have several effects, depending on the level of perfusion. Increased blood perfusion was observed to result in swelling of the toe tip, which could act to increase contact area of the toe tip as it expands proximally or laterally on a smooth substrate, or act to lock it into a fissure in a roughened substrate. Blood moving out of the toe tip may act to increase the flaccidity of the toe tip structures, allowing them to adhere more successfully because the pad is able to conform more closely to rough surfaces, as has been suggested for other animals (Barnes 2007; Barnes et al. 2011; Dai and Gorb 2009; Federle et al. 2006). Flexible surfaces, such as expandable toe tips, can enhance frictional and adhesive contact due to their increased compliance, permitting greater area of contact (Persson et al. 2005; Peressadko, Hosoda, and Persson 2005). Other observed anatomical features such as the pleating of the integument during stepping, the presence of the mucous glands on the dorsal and dorsolateral surface of the phalanx, and the recurved distal phalanx itself may play a role in enhancing attachment through increasing friction, wet adhesion, or interlocking with the substrate during landing from a jump.

### 4.6 Future Directions

We will close with future directions for testing how mechanical control of the digital vascular system in *Aneides* might function to assist locomotion, informed by what we know about digital vascular systems in other species. In tree frog toe pads, digital flexion causes the penultimate phalanx to press down on the sinus, leading to increased pressure driving the pad onto and facilitating close contact with the substrate and resulting in increased adhesion or friction. Furthermore, it is evident that tree frogs can alter pad stiffness according to prevailing circumstances, since blood pressure is under physiological control (Barnes et al. 2011).

The data presented here, which suggest predictable changes in the amount of blood within the chambers of the sinus upon surface detachment, pose a similar scenario in which the toe tips of *A. vagrans* might vary in stiffness according to blood pressure. The timing of increased blood perfusion near toe off suggests a role in detachment. Thus, in contrast to the attachment mechanisms described for treefrogs, we hypothesize *A. vagrans* fills the sinuses at the toe tips to disengage (rather than engage) the toes in surface attachment. Under this scenario, blood would engorge the toe tip such that the radius of curvature of the tip is increased and thus the area of contact with the substrate is decreased, reducing the overall detachment forces required. This scenario aligns with the empirical data presented above, and is similar to the phenomena seen in other climbing organisms (Barnes et al. 2011; Dai and Gorb 2009; Dening et al. 2014; Flammang and Kenaley 2017). Importantly, that the digital vascular system of *A. vagrans* works via fluid pressure differences implies that the toe tips are deformable along multiple axes at any one time, facilitating compliance with the substrate. The role of the recurved distal end of the terminal phalanx is worthy of biomechanical analysis as well, and determination of its spatulate or bifurcate shape. We hypothesize that fluid‐pressure changes within the toe tips assist compliance with and attachment to the substrate as well as release from it.

Our live‐animal experiment was limited in that we were unable to calculate the exact rate of blood cell flow (m/s); future live‐animal experiments exploring the digital vascular system of salamanders should consider employing a camera capable of recording videos at frame rates high enough to point‐track individual red blood cells to quantify the fluid dynamics underlying such rapid circulation and vessel dilation. These functional hypotheses await formal testing across a broader range of climbing and scansorial salamander species, but if supported in future experiments, could motivate new bioinspired designs (e.g., Geckskin, Somerville, MA, USA) (Afferante et al. 2016).

## 5 Conclusions

Arboreal wandering salamanders (*A. vagrans*) possess a digital vascular system similar to that of other members of their genus. Histological investigations along with live‐animal video observations suggest that blood rapidly flows into the digital sinus, functionally expanding the surface of the toe tip that is breaking contact with the substrate. Our findings suggest the possibility that this is a morphological adaptation for clinging, climbing, or other mode of arboreal locomotion that could benefit from fine‐tuned control of individual toe‐tips, such as landing on fern mats after falling or gliding. Furthermore, we posit that the digital vascular system may allow for rapid mechanical adjustments of blood volume in the toes, as has been suggested for other skilled arboreal locomotors, such as Tokay geckos. The functional anatomy of the digital vascular system of climbing salamanders has potential implications for bioinspired designs.

## Author Contributions

Conceptualization: C.E.B., W.G., and N.L.S. Methodology: all authors. Investigation: all authors. Software: all authors. Formal analysis: all authors. Validation: C.E.B., MK.O., and N.L.S. Writing–original draft: C.E.B., O.M.H., and MK.O. Writing–review and editing: all authors. Visualization: all authors.

## Conflicts of Interest

The authors declare no conflicts of interest.

### 1 Peer Review

The peer review history for this article is available at https://www.webofscience.com/api/gateway/wos/peer-review/10.1002/jmor.70026.

## Supporting information


**Movie 1. Lateral view of blood circulating through the digital vascular system of** ***Aneides vagrans***, **with focus pulled to the large sinus.** Video plays back at 1000x normal speed, first playing forwards, then in reverse, then looping in that fashion three times before comparing screenshots of the vascular network when empty and full (same comparison as in Figure 5).

**Movie 2. Ventral view of blood circulating through the digital vascular system of** ***Aneides vagrans***. This view shows the connection between the sinus chambers that was also observed in histological preparations of the toes.

**Movie 3. Ventral view of pleats in the epidermis of the square‐shaped toe tip in** ***Aneides vagrans***. The pleats appear to dissipate and then reform as the toe tip moves, that being especially evident at toe off.

**Supplemental Figure 1. Photomicrograph of a 0.90 μm cross‐section of Digit III of the hindfoot of** ***Aneides vagrans***. This slide was stained with toluidine blue. Scale bar is 100 μm.

## Data Availability

The data that support the findings of this study are openly available in Zenodo online repository at 10.5281/zenodo.13371829. This data can also be accessed by navigating to Zenodo.org and searching ‘*Aneides vagrans* toes.’ All data are also available upon request to the corresponding author.

## References

[jmor70026-bib-0001] Adams, D. C. , D. Korneisel , M. Young , and A. Nistri . 2017. “Natural History Constrains the Macroevolution of Foot Morphology in European Plethodontid Salamanders.” American Naturalist 190, no. 2: 292–297. 10.1086/692471. 28731800

[jmor70026-bib-0002] Adams, D. C. , and A. Nistri . 2010. “Ontogenetic Convergence and Evolution of Foot Morphology in European Cave Salamanders (Family: Plethodontidae).” BMC Evolutionary Biology 10: 216. 10.1186/1471-2148-10-216. 20637087 PMC2927916

[jmor70026-bib-0003] Afferante, L. , L. Heepe , K. Casdorff , S. Gorb , and G. Carbone . 2016. “A Theoretical Characterization of Curvature Controlled Adhesive Properties of Bio‐Inspired Membranes.” Biomimetics 1, no. 1: 3. 10.3390/biomimetics1010003.

[jmor70026-bib-0004] Aretz, J. , C. E. Brown , and S. M. Deban . 2021. “Vertical Locomotion in the Arboreal Salamander *Aneides Vagrans* .” Zoology 316: 72–79. 10.1111/jzo.12934.

[jmor70026-bib-0005] Baken, E. K. , and D. C. Adams . 2019. “Macroevolution of Arboreality in Salamanders.” Ecology and Evolution 9, no. 12: 7005–7016. 10.1002/ece3.5267. 31380029 PMC6662381

[jmor70026-bib-0006] Baken, E. K. , and M. K. O'Donnell . 2021. “Clinging Ability Is Related to Particular Aspects of Foot Morphology in Salamanders.” Ecology and Evolution 11, no. 16: 11000–11008. 10.1002/ece3.7888. 34429897 PMC8366850

[jmor70026-bib-0007] Barnes, W. J. P. 2007. “Functional Morphology and Design Constraints of Smooth Adhesive Pads.” MRS Bulletin 32: 479–485. 10.1557/mrs2007.81.

[jmor70026-bib-0008] Barnes, W. J. P. , P. J. P. Goodwyn , M. Nokhbatolfoghahai , and S. N. Gorb . 2011. “Elastic Modulus of Tree Frog Adhesive Toe Pads.” Journal of Comparative Physiology A 197: 969–978. 10.1007/s00359-011-0658-1. PMC3176399 21667266

[jmor70026-bib-0009] Barnes, W. J. P. , C. Oines , and J. M. Smith . 2006. “Whole Animal Measurements of Shear and Adhesive Forces in Adult Tree Frogs: Insights Into Underlying Mechanisms of Adhesion Obtained From Studying the Effects of Size and Scale.” Journal of Comparative Physiology A 192: 1179–1191. 10.1007/s00359-006-0146-1. 16924504

[jmor70026-bib-0010] Blankers, T. , D. C. Adams , and J. J. Wiens . 2012. “Ecological Radiation With Limited Morphological Diversification in Salamanders.” Journal of Evolutionary Biology 25, no. 4: 634–646. 10.1111/j.1420-9101.2012.02458.x. 22268991

[jmor70026-bib-0011] Brown, A. G. 1972. “ *Responses to Problems of Water and Electrolyte Balance by Salamanders (Genus Aneides) From Different Habitats* .” PhD diss., Berkeley: University of California.

[jmor70026-bib-0012] Brown, C. E. , and S. M. Deban . 2020. “Jumping in Arboreal Salamanders: A Possible Tradeoff Between Takeoff Velocity and In‐Air Posture.” Zoology 138: 125724. 10.1016/j.zool.2019.125724. 31951970

[jmor70026-bib-0013] Brown, C. E. , E. A. Sathe , R. Dudley , and S. M. Deban . 2022. “Aerial Maneuvering by Plethodontid Salamanders Spanning an Arboreality Gradient.” Journal of Experimental Biology 225, no. 20: jeb244598. 10.1242/jeb.244598. 36111422

[jmor70026-bib-0014] Burggren, W. , and R. Moallf . 1984. “Active' Regulation of Cutaneous Exchange by Capillary Recruitment in Amphibians: Experimental Evidence and a Revised Model for Skin Respiration.” Respiration Physiology 55, no. 3: 379–392. 10.1016/0034-5687(84)90059-8. 6429804

[jmor70026-bib-0015] Cartmill, M. 1985. “Climbing.” In Functional Vertebrate Morphology, edited by M. Hildebrand , D. M. Bramble , K. F. Liem , and D. B. Wake , 73–88. Cambridge: Harvard University Press.

[jmor70026-bib-0016] Dai, Z. , and S. Gorb . 2009. “Contact Mechanics of Pad of Grasshopper (Insecta: Orthoptera) by Finite Element Methods.” Chinese Science Bulletin 54, no. 4: 549–555. 10.1007/s11434-009-0088-4.

[jmor70026-bib-0017] Dening, K. , L. Heepe , L. Afferrante , G. Carbone , and S. N. Gorb . 2014. “Adhesion Control by Inflation: Implications From Biology to Artificial Attachment Device.” Applied Physics A 116, no. 2: 567–573. 10.1007/s00339-014-8504-2.

[jmor70026-bib-0018] Diefenbacher, E. H. 2008a. “ *Comparing Digit Morphology of an Arboreal Salamander With Potential Competitors* .” PhD diss., Marshall University Digital Scholar. https://mds.marshall.edu/etd/406.

[jmor70026-bib-0019] Diefenbacher, E. H. 2008b. “ *Aneides aeneus* Digit Morphology.” Herpetological Review 39, no. 4: 454–455.

[jmor70026-bib-0020] Dixon, J. R. , and A. G. Kluge . 1964. “A New Gekkonid Lizard Genus From Australia.” Copeia 1964: 174.

[jmor70026-bib-0021] Dudley, R. , G. Byrnes , S. P. Yanoviak , B. J. Borrell , R. Brown , and J. A. McGuire . 2007. “Gliding and the Functional Origins of Flight: Biomechanical Novelty or Necessity?” Annual Review of Ecology, Evolution, and Systematics 38: 179–201. 10.1146/annurev.ecolsys.37.091305.110014.

[jmor70026-bib-0022] Dudley, R. , and S. P. Yanoviak . 2011. “Animal Aloft: The Origins of Aerial Behavior and Flight.” Integrative and Comparative Biology 51, no. 6: 926–936. 10.1093/icb/icr002. 21558180

[jmor70026-bib-0023] Federle, W. , W. J. P. Barnes , W. Baumgartner , P. Drechsler , and J. M. Smith . 2006. “Wet But Not Slippery: Boundary Friction in Tree Frog Adhesive Toe Pads.” Journal of the Royal Society Interface 3, no. 10: 689–697. 10.1098/rsif.2006.0135. 16971337 PMC1664653

[jmor70026-bib-0024] Flammang, B. E. , and C. P. Kenaley . 2017. “Remora Cranial Vein Morphology and Its Functional Implications for Attachment.” Scientific Reports 7, no. 1: 5914. 10.1038/s41598-017-06429-z. 28725032 PMC5517627

[jmor70026-bib-1024] Floyd, A. D. 1990. “Morphology and the Art of Tissue Analysis.” Laboratory Leader 5: 3–6.

[jmor70026-bib-0025] Green, D. M. 1979. “Treefrog Toe Pads: Comparative Surface Morphology Using Scanning Electron Microscopy.” Canadian Journal of Zoology 57, no. 10: 2033–2046. 10.1139/z79-268.

[jmor70026-bib-0026] Green, D. M. , and P. Alberch . 1981. “Interdigital Webbing and Skin Morphology in the Neotropical Salamander Genus *Bolitoglossa* (Amphibia; Plethodontidae).” Journal of Morphology 170, no. 3: 273–282. 10.1002/jmor.1051700302. 30119588

[jmor70026-bib-0027] Hanna, C. S. , C. Alihosseini , H. M. Fischer , E. C. Davoli , and M. C. Granatosky . 2022. “Are They Arboreal? Climbing Abilities and Mechanics in the Red‐Backed Salamander (*Plethodon Cinereus*).” Journal of Experimental Zoology Part A: Ecological and Integrative Physiology 337, no. 3: 238–249. 10.1002/jez.2561. 34752693

[jmor70026-bib-0028] Humphreys, R. K. , and G. D. Ruxton . 2019. “Dropping to Escape: A Review of an Under‐Appreciated Antipredator Defence.” Biological Reviews 94, no. 2: 575–589. 10.1111/brv.12466. 30298642

[jmor70026-bib-1028] Jackman, T. R. 1998. “Molecular and Historical Evidence for the Introduction of Clouded Salamanders (Genus Aneides) to Vancouver Island, British Columbia, Canada, From California.” Canadian Journal of Zoology 76, no. 8: 1570–1580.

[jmor70026-bib-0029] Jaekel, M. , and D. B. Wake . 2007. “Developmental Processes Underlying the Evolution of a Derived Foot Morphology in Salamanders.” Proceedings of the National Academy of Sciences 104, no. 51: 20437–20442. 10.1073/pnas.0710216105. PMC2154449 18077320

[jmor70026-bib-0030] Jusufi, A. , Y. Zeng , R. J. Full , and R. Dudley . 2011. “Aerial Righting Reflexes in Flightless Animals.” Integrative and Comparative Biology 51, no. 6: 937–943. 10.1093/icb/icr114. 21930662

[jmor70026-bib-0031] Kays, R. , and A. Allison . 2001. “Arboreal Tropical Forest Vertebrates: Current Knowledge And Research Trends.” In Tropical Forest Canopies: Ecology and Management. Forestry Sciences, edited by K. E. Linsenmair , A. J. Davis , B. Fiala , and M. R. Speight , 109–120. Dordrecht: Springer. 10.1007/978-94-017-3606-0_9.

[jmor70026-bib-0032] Krogh, A. , G. A. Harrop , and P. B. Rehberg . 1922. “Studies on the Physiology of Capillaries: III. The Innervation of the Blood Vessels in the Hind Legs of the Frog.” Journal of Physiology 56, no. 3–4: 179–189. 10.1113/jphysiol.1922.sp002000. 16993560 PMC1405392

[jmor70026-bib-0033] Langowski, J. K. A. , D. Dodou , M. Kamperman , and J. L. van Leeuwen . 2018. “Tree Frog Attachment: Mechanisms, Challenges, and Perspectives.” Frontiers in Zoology 15, no. 1: 32. 10.1186/s12983-018-0273-x. 30154908 PMC6107968

[jmor70026-bib-0034] McEntire, K. D. 2016. “Arboreal Ecology of Plethodontidae: A Review.” Copeia 104, no. 1: 124–131. 10.1643/OT-14-214.

[jmor70026-bib-0036] Noble, G. K. 1925. “The Integumentary, Pulmonary, and Cardiac Modifications Correlated With Increased Cutaneous Respiration in the Amphibia: A Solution of the ‘Hairy Frog’ Problem.” Journal of Morphology 40, no. 2: 341–416. 10.1002/jmor.1050400206.

[jmor70026-bib-0037] O'Donnell, M. K. , and S. M. Deban . 2020a. “Cling Performance and Surface Area of Attachment in Plethodontid Salamanders.” Journal of Experimental Biology 223, no. 17: jeb211706. 10.1242/jeb.211706. 32675231

[jmor70026-bib-0038] O'Donnell, M. K. , and S. M. Deban . 2020b. “The Effects of Roughness and Wetness on Salamander Cling Performance.” Integrative and Comparative Biology 60, no. 4: 840–851. 10.1093/icb/icaa110. 32687157

[jmor70026-bib-0039] Peressadko, A. G. , N. Hosoda , and B. N. J. Persson . 2005. “Influence of Surface Roughness on Adhesion Between Elastic Bodies.” Physical Review Letters 95: 124301. 10.1103/PhysRevLett.95.124301. 16197078

[jmor70026-bib-0040] Persson, B. N. , O. Albohr , U. Tartaglino , A. I. Volokitin , and E. Tosatti . 2005. “On the Nature of Surface Roughness With Application to Contact Mechanics, Sealing, Rubber Friction, and Adhesion.” Journal of Physics. Condensed Matter: An Institute of Physics Journal 17, no. 1: 1. 10.1088/0953-8984/17/1/R01. 21690662

[jmor70026-bib-0041] Petranka, J. W. 1998. Salamanders of the United States and Canada. Washington, London: Smithsonian Institution Press.

[jmor70026-bib-0042] Rasband, W. S. 1997–2023. ImageJ. U.S. National Institutes of Health. https://imagej.nih.gov/ij/.

[jmor70026-bib-0043] Raygoza, P . 2018. Determining the Morphology of the Expanded Toe Tips of the Lungless Salamander, Aneides lugubris. Spokane, WA, USA: Research Concentration paper for Gonzaga University.

[jmor70026-bib-0044] RStudio Team . (2020). RStudio: Integrated Development for R. RStudio. http://www.rstudio.com/.

[jmor70026-bib-0035] Ritter, W. E. , and L. Miller . 1899. “A Contribution to the Life History of *Autodax lugubris* Hallow., a Californian Salamander.” American Naturalist 33, no. 393: 691–704. https://www.journals.uchicago.edu/doi/abs/10.1086/277412.

[jmor70026-bib-0045] Russell, A. P. 1981. “Descriptive and Functional Anatomy of the Digital Vascular System of the Tokay, *Gekko gecko* .” Journal of Morphology 169, no. 3: 293–323. 10.1002/jmor.1051690305. 30114860

[jmor70026-bib-0046] Salvidio, S. , F. Crovetto , and D. C. Adams . 2015. “Potential Rapid Evolution of Foot Morphology in Italian Plethodontid Salamanders (*Hydromantes strinatii*) Following the Colonization of an Artificial Cave.” Journal of Evolutionary Biology 28, no. 7: 1403–1409. 10.1111/jeb.12654. 25975804

[jmor70026-bib-0047] Scholz, I. , W. J. P. Barnes , J. M. Smith , and W. Baumgartner . 2009. “Ultrastructure and Physical Properties of an Adhesive Surface, the Toe Pad Epithelium of the Tree Frog, *Litoria caerulea* White.” Journal of Experimental Biology 212, no. 2: 155–162. 10.1242/jeb.019232. 19112133 PMC2720997

[jmor70026-bib-0048] Sillett, S. C. 1999. “Tree Crown Structure and Vascular Epiphyte Distribution in *Sequoia sempervirens* Rain Forest Canopies.” Selbyana 20: 76–97. https://www.jstor.org/stable/41760010.

[jmor70026-bib-0049] Smith, J. M. , W. J. P. Barnes , J. R. Downie , and G. D. Ruxton . 2006. “Structural Correlates of Increased Adhesive Efficiency With Adult Size in the Toe Pads of Hylid Tree Frogs.” Journal of Comparative Physiology A 192: 1193–1204. 10.1007/s00359-006-0151-4. 16960739

[jmor70026-bib-0050] Spickler, J. C. , S. C. Sillett , S. B. Marks , and H. H. Welsh Jr . 2006. “Evidence of a New Niche for a North American Salamander: *Aneides vagrans* Residing in the Canopy of Old‐Growth Redwood Forest.” Herpetological and Conservation Biology 1: 16–27.

[jmor70026-bib-1050] Staub, N. L. , and J. Paladin . 1997. “The Presence of Modified Granular Glands in Male and Female Aneides Lugubris.” Herpetologica 53: 339–344.

[jmor70026-bib-0051] Stebbins, R. C. 2003. A Field Guide to Western Reptiles and Amphibians, 3rd ed. Boston, New York: Houghton Mifflin Press.

[jmor70026-bib-0052] Wake, D. B. 1963. “Comparative Osteology of the Plethodontid Salamander Genus *Aneides* .” Journal of Morphology 113, no. 1: 77–118. 10.1002/jmor.1051130106. 13998371

[jmor70026-bib-0053] Wake, T. A. , D. B. Wake , and M. H. Wake . 1983. “The Ossification Sequence of *Aneides lugubris*, With Comments on Heterochrony.” Journal of Herpetology 17, no. 1: 10–22. 10.2307/1563775.

[jmor70026-bib-0054] Wang, S. , M. Li , W. Huang , and X. Wang . 2016. “Sticking/Climbing Ability and Morphology Studies of the Toe Pads of Chinese Fire Belly Newt.” Journal of Bionic Engineering 13, no. 1: 115–123. 10.1016/S1672-6529(14)60165-7.

