# Supplementary figures and images for: Vascular and Osteological Morphology of Expanded Digit Tips Suggests Specialization in the Wandering Salamander (Aneides vagrans)
Source: J Morphol. 2025 Jan 8;286(1):e70026. doi: 10.1002/jmor.70026 (PMC11711880; doi:10.1002/jmor.70026)

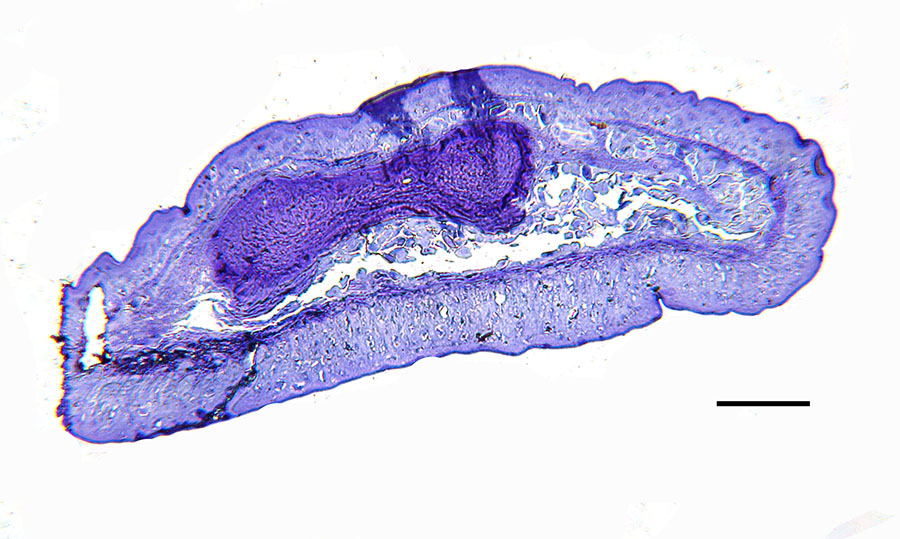

Supplement: Supplementary file 4 — Supplemental Figure 1. Photomicrograph of a 0.90 μm cross‐section of Digit III of the hindfoot of Aneides vagrans . This slide was stained with toluidine blue. Scale bar is 100 μm. [file JMOR-286-e70026-s003.jpg]
